# Supplementary material for: Genome-wide discovery of hidden genes mediating known drug-disease association using KDDANet
Source: NPJ Genom Med. 2021 Jun 15;6:50. doi: 10.1038/s41525-021-00216-6 (PMC8206141; doi:10.1038/s41525-021-00216-6)
Supplement: Supplementary file 2 — Reporting Summary [file 41525_2021_216_MOESM2_ESM.pdf]

nature research

Corresponding author(s): HUA YU
Last updated by author(s): Mar 13, 2021

Reporting Summary

Nature Research wishes to improve the reproducibility of the work that we publish. This form provides structure for consistency and transparency in reporting. For further information on Nature Research policies, see our Editorial Policies and the Editorial Policy Checklist.

Statistics

For all statistical analyses, confirm that the following items are present in the figure legend, table legend, main text, or Methods section.

n/a	Confirmed	
☐	☒	The exact sample size (*n*) for each experimental group/condition, given as a discrete number and unit of measurement
☐	☒	A statement on whether measurements were taken from distinct samples or whether the same sample was measured repeatedly
☐	☒	The statistical test(s) used AND whether they are one- or two-sided<br>*Only common tests should be described solely by name; describe more complex techniques in the Methods section.*
☒	☐	A description of all covariates tested
☐	☒	A description of any assumptions or corrections, such as tests of normality and adjustment for multiple comparisons
☐	☒	A full description of the statistical parameters including central tendency (e.g. means) or other basic estimates (e.g. regression coefficient) AND variation (e.g. standard deviation) or associated estimates of uncertainty (e.g. confidence intervals)
☐	☒	For null hypothesis testing, the test statistic (e.g. *F*, *t*, *r*) with confidence intervals, effect sizes, degrees of freedom and *P* value noted<br>*Give P values as exact values whenever suitable.*
☒	☐	For Bayesian analysis, information on the choice of priors and Markov chain Monte Carlo settings
☒	☐	For hierarchical and complex designs, identification of the appropriate level for tests and full reporting of outcomes
☐	☒	Estimates of effect sizes (e.g. Cohen's *d*, Pearson's *r*), indicating how they were calculated

Our web collection on statistics for biologists contains articles on many of the points above.

Software and code

Policy information about availability of computer code

Data collection

No computer code was used to collect the data

Data analysis

RNA-seq reads were trimmed using Trimmomatic software with the following parameters "ILLUMINACLIP:TruSeq3-PE.fa:2:30:10 LEADING:3 TRAILING:3 SLIDINGWINDOW:4:15 MINLEN:36" (Version 0.36), and were further quality-filtered using FASTX Toolkit's fastq_quality_trimmer command (Version 0.0.13) with the minimum quality score 20 and minimum percent of 80% bases that had a quality score larger than this cutoff value. The high-quality reads were mapped to the hg38 genome by HISAT2, a fast and sensitive spliced alignment program for mapping RNA-seq reads, with -dta paramenter. PCR duplicate reads were removed using Picard tools and only uniquely mapped reads were kept for further analysis. The expression levels of genes were calculated by StringTie (Version v1.3.4d) with -e -B -G parameters using Release 29 (GRCh38.p12) gene annotations downloaded from GENCODE data portal (https://www.gencodegenes.org/). To obtain comparable expression abundance estimation for each gene, reads mapped to hg38 were counted as FPKM (Fragments Per Kilobase Of Exon Per Million Fragments Mapped) based on their genome locations. Differential expression analysis of genes was performed by DESeq2 using the reads count matrix produced from a python script "prepDE.py" provided in StringTie website (http://ccb.jhu.edu/software/stringtie/).

The source codes of KDDANet software are freely available at https://github.com/huayu1111/KDDANet.

All custom codes were generated using Perl, Python or R, and can be available upon request.

For manuscripts utilizing custom algorithms or software that are central to the research but not yet described in published literature, software must be made available to editors and reviewers. We strongly encourage code deposition in a community repository (e.g. GitHub). See the Nature Research guidelines for submitting code & software for further information.

Data

Policy information about availability of data

All manuscripts must include a data availability statement. This statement should provide the following information, where applicable:

- Accession codes, unique identifiers, or web links for publicly available datasets
- A list of figures that have associated raw data
- A description of any restrictions on data availability

The KDDANet resulting subnetworks, supporting the conclusions of this article are available in our online web server (http://www.kddanet.cn). Additional datasets are described in the Methods section and are presented in the Dataset 1, Dataset2, Dataset 3 and Dataset 4.

Field-specific reporting

Please select the one below that is the best fit for your research. If you are not sure, read the appropriate sections before making your selection.

☒ Life sciences ☐ Behavioural & social sciences ☐ Ecological, evolutionary & environmental sciences

For a reference copy of the document with all sections, see nature.com/documents/nr-reporting-summary-flat.pdf

Life sciences study design

All studies must disclose on these points even when the disclosure is negative.

Sample size	Five different types of gene networks, HumanNet, HINT+HI2012, iRefIndex, MultiNet and STRINGv10.<br>4861 drugs.<br>2196 known drug target genes.<br>1441 diseases.<br>1521 disease-related genes.<br>53124 drug-disease associations.
Data exclusions	We selected 4861 drugs with at least one known target that was contained in the gene networks for further analysis. In total, 2196 Known Drug Target Genes (KDTGs) included in the gene networks were connected to these drugs by 12014 interactions. Known Disease Related Genes (KDRGs) and classification of diseases were obtained by manually collecting Human Disease Network (HDN) from the previous study of Human Disease Network (HDN) 35 and DisGeNET v5.0 database. We focused on 1441 diseases with at least one related gene which was included in the gene networks for our study. In total, 16712 associations link these diseases to 1521 genes which were exist in the gene networks. The KDDAs were extracted from Comparative Toxicogenomics Database 12. In this study, 53124 KDDAs were analyzed in which the drug had at least one target gene and the disease had at least one related gene contained in HumanNet.
Replication	N/A, no replications were taken
Randomization	Permutation test
Blinding	N/A, no measurements were taken

Reporting for specific materials, systems and methods

We require information from authors about some types of materials, experimental systems and methods used in many studies. Here, indicate whether each material, system or method listed is relevant to your study. If you are not sure if a list item applies to your research, read the appropriate section before selecting a response.

Materials & experimental systems

n/a	Involved in the study	
☒	☐	Antibodies
☒	☐	Eukaryotic cell lines
☒	☐	Palaeontology and archaeology
☒	☐	Animals and other organisms
☒	☐	Human research participants
☒	☐	Clinical data
☒	☐	Dual use research of concern

Methods

n/a	Involved in the study	
☒	☐	ChIP-seq
☒	☐	Flow cytometry
☒	☐	MRI-based neuroimaging
